# Three-year sublingual immunotherapy for HDM-induced allergic rhinoconjunctivitis: sustained ocular symptom improvement

**DOI:** 10.3389/fmed.2026.1834048

**Published:** 2026-04-30

**Authors:** Chenggang Liu, Hong Jiang, Yu Long, Feng Jiang, Lihong Chen

**Affiliations:** 1 Department of Ophthalmology, Yongchuan Hospital of Chongqing Medical University (The Fifth Clinical College of Chongqing Medical University), General Practice School of Chongqing Medical University, Chongqing, China; 2 Department of Otolaryngology Head and Neck Surgery, Yongchuan Hospital of Chongqing Medical University (The Fifth Clinical College of Chongqing Medical University), General Practice School of Chongqing Medical University, Chongqing, China

**Keywords:** allergic rhinoconjunctivitis, efficacy, house dust mite, sublingual immunotherapy, symptom improvement

## Abstract

**Background:**

Allergic conjunctivitis (AC) frequently coexists with allergic rhinitis (AR), together known as allergic rhinoconjunctivitis (ARC), but ocular symptoms are often underemphasized in efficacy assessments using composite scores. Therefore, this study aimed to isolate and quantify the therapeutic effect of house dust mite sublingual immunotherapy (HDM-SLIT) on ocular symptoms, providing targeted evidence for managing AC in HDM-sensitized patients.

**Methods:**

The complete clinical data of 150 patients with ARC aged 6–59 years who started HDM-SLIT for 3 years between March 2020 and May 2022 were retrospectively analyzed. The level of the total nasal symptom score (TNSS), total ocular symptom score (TOSS), total medication score (TMS), combined score of medication and rhinoconjunctivitis symptoms (CSMRS), the visual analog scale (VAS) score, and rhinoconjunctivitis quality of life questionnaire (RQLQ) were evaluated at baseline, 1, 2, and 3 years of treatment. Additionally, the occurrence of adverse events (AEs) was used to evaluate its safety.

**Results:**

Compared with baseline, significant improvements in TNSS, TOSS, TMS, CSMRS, and VAS scores were observed after 1, 2, and 3 years of SLIT (all *p* < 0.001). Compared with 1-year results, TNSS, CSMRS, and VAS scores showed further improvements at 2 and 3 years (all *p* < 0.001). RQLQ scores improved significantly across all domains and age groups (6–11 years, 12–17 years, and ≥18 years groups) at all time points (all *p* < 0.001). Subgroup analysis revealed no significant difference in efficacy between the children and adult groups. Throughout the 3-year treatment period, no serious systemic AEs were reported.

**Conclusion:**

Three-year HDM-SLIT yields significant, sustained, and progressive improvements in clinical outcomes and quality of life in patients with ARC, along with significant improvements in ocular symptoms and a favorable safety profile.

## 1 Introduction

Allergic rhinitis (AR) frequently coexists with allergic conjunctivitis (AC) (1). Epidemiological studies have demonstrated a close link between these conditions, with ocular symptoms reported in 32 to 59% of patients with AR in China (2, 3). This concomitant nasal and ocular manifestation arises not only from anatomical continuity—the eyes serving as an extension of the airways—but also from shared T-helper 2 (Th2)-type immune-inflammatory pathways and the “naso-ocular reflex,” thus giving rise to the clinical entity known as allergic rhinoconjunctivitis (ARC) (1, 4).

House dust mites (HDM) are among the most predominant allergens triggering respiratory allergic diseases in China. Investigations have revealed that dust mite sensitization is remarkably prevalent in patients with allergic diseases, with positive rates for *Dermatophagoides farinae* (*Der f*) and *Dermatophagoides pteronyssinus* (*Der p*) reaching 59 and 57.6%, respectively (5, 6)—rates significantly exceeding those of other inhalant allergens. The management of HDM-induced allergic diseases is based on a four-pronged approach encompassing allergen avoidance, symptomatic pharmacotherapy, allergen immunotherapy (AIT), and patient education (7). Among these, AIT is considered the only treatment modality that may modify the natural course of allergic diseases (8). Sublingual immunotherapy (SLIT), in particular, is recognized as a safer option and is widely used in clinical practice for HDM-sensitized patients (9, 10).

Numerous studies have validated the clinical efficacy and safety of AIT for AR or ARC (1, 6, 11, 12). However, the independent impact of ocular symptoms (such as itching, redness, and tearing) on patients’ quality of life, sleep, and daily concentration is often underestimated, as they are typically regarded as mere manifestations of rhinitis (4). Consequently, the efficacy evaluation of most interventions mainly focuses on improving nasal symptoms, or assesses both nasal and ocular symptoms as a combined “rhinoconjunctivitis” endpoint indicator (1). Thus, studies specifically evaluating ocular symptom improvement following HDM-SLIT remain scarce. Several review studies indicated that SLIT can significantly reduce total ocular symptom scores and individual scores for itching, redness, and tearing; however, the evidence base remains limited by issues such as heterogeneity, underscoring the need for more rigorously designed studies to validate these findings (11, 12). Therefore, in this study, we aimed to isolate and quantify the therapeutic effect of HDM-SLIT on ocular symptoms, thereby providing targeted evidence for the management of AC in patients with HDM sensitization.

## 2 Methods

### 2.1 Patients

**Figure 1:**
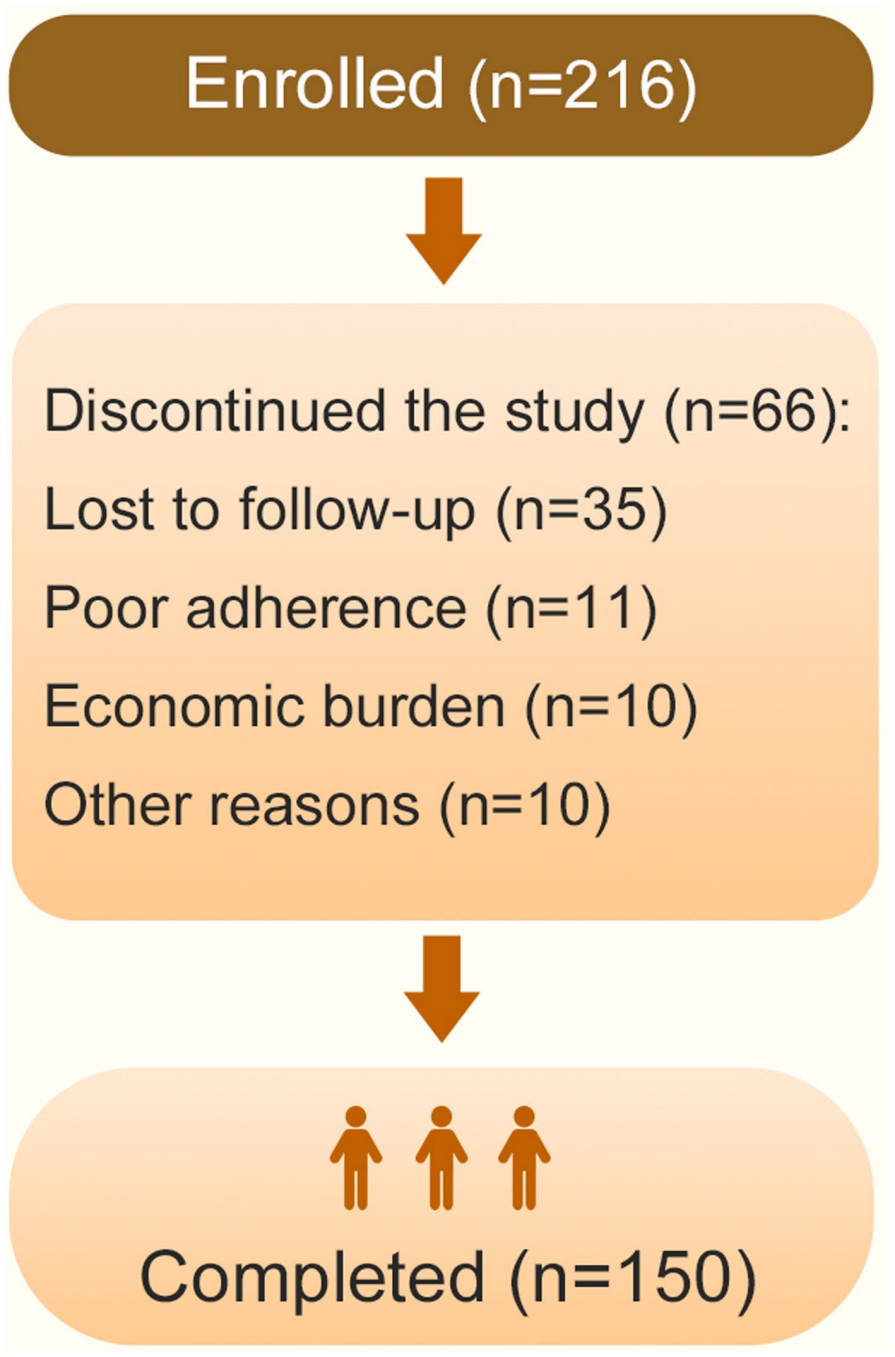
Flowchart of the number of study subjects.

A total of 216 patients aged 6–59 years with ARC who presented to Yongchuan hospital of Chongqing medical university from March 2020 to May 2022 were enrolled. Finally, 150 patients completed 3 years of SLIT treatment were analyzed in our study (Figure 1). The treatment criteria of SLIT included: (1) patients were diagnosed with moderate-to-severe AR with AC according to the Chinese guidelines for diagnosis and treatment of allergic rhinitis (2022 revision) (13) and the Allergic Rhinitis and its Impact on Asthma (ARIA) 2008 update (14); (2) patients have a clinical history of mite allergy and confirmed sensitization to *Der f* and/or *Der p* by a positive skin prick test (Zhejiang Wolwo Bio-Pharmaceutical Co., Ltd., Zhejiang, China); Exclusion criteria included: (i) severe or uncontrolled asthma; (ii) systemic immune diseases; (iii) use of β-blockers or angiotensin-converting enzyme inhibitors; (iv) pregnancy or lactation; (v) planning pregnancy within one year.

The present study was approved by the Ethics Committee of Yongchuan hospital of Chongqing medical university (Approval No. KLS-2024-41) and was conducted in accordance with the Declaration of Helsinki and the Guidelines for Good Clinical Practice. Written informed consent was obtained from all patients or their legal guardians prior to participation.

### 2.2 Treatment regimen

In this study, all patients were administered *Der f* sublingual drops (Chanllergen; Zhejiang Wolwo Bio-Pharmaceutical Co., Ltd., Hangzhou, China) daily for three years according to the manufacturer’s instructions. The allergen extract is a standardized preparation of *Der f* with total protein concentrations of 1, 10, 100, 333, and 1,000 μg/mL for bottles No. 1 to No. 5, respectively, as determined by the bicinchoninic acid protein assay. The treatment schedule included an up-dosing phase and a maintenance phase. The specific administration regimen was as follows: All patients, regardless of age, followed the same schedule for the first 3 weeks, patients were instructed to take drops No. 1, drops No. 2, and drops No. 3 in weeks 1, 2, and 3, respectively. Within each week, the drops were administered in the order of 1, 2, 3, 4, 6, 8, and 10 drops from day 1 to day 7. From week 4 onward, children patients (aged <14 years) entered the maintenance phase, taking three drops of No. 4 daily for the remainder of the treatment course. For adult patients (aged ≥14 years), from week 4 to 5 continued the up-dosing phase with No. 4 at a dose of 3 drops daily. From week 6 onward, these patients entered the maintenance phase, taking 2 drops of No. 5 daily until the end of the three-year treatment course. All doses were self-administered sublingually at the same time each day and held for 1–3 min before swallowing. The first dose was administered under medical supervision, and patients were observed for at least 30 min thereafter.

### 2.3 Clinical efficacy

At baseline, nasal and ocular symptoms, medication use, the Visual Analog Scale (VAS) score, Rhinoconjunctivitis Quality of Life Questionnaire (RQLQ) score, and adverse events (AEs) were recorded before the treatment. During the 3-year treatment course, patients (or their guardians) were scheduled for follow-up visits either at the hospital or by telephone. Symptom and medication use data were collected using daily diary cards, in which patients recorded their symptoms and medication intake over the preceding week. The average scores were then calculated to obtain the final scores at each time point. Efficacy was evaluated at 1, 2, and 3 years of treatment. Safety was analyzed based on the AEs recorded during the study period. All AEs were addressed under the recommendation of the physicians.

The Total Nasal Symptom Score (TNSS) was calculated as the sum of the scores for nasal itching, congestion, sneezing, and rhinorrhea. The Total Ocular Symptom Score (TOSS) was defined as the sum of the scores for itchy/gritty feeling/red eyes and watery eyes. All symptoms were assessed using a 0–3 scale (0 = no symptoms, 1 = mild, 2 = moderate, 3 = severe) (6, 15–18). The Total Medication Score (TMS) was defined as follows: 0 = no medication use; 1 = H1-antihistamines (AH) only; 2 = intranasal corticosteroids (INS, with or without AH); 3 = oral corticosteroids (OCS, with or without AH) (15–18). The Combined Score of Medication and rhinoconjunctivitis Symptoms (CSMRS) was calculated as: CSMRS = (TNSS + TOSS)/6 + TMS (18). The VAS, ranging from 0 (no symptoms) to 10 (severe symptoms), was used to quantify allergic symptom burden (19).

The RQLQ score (0–6 point) reflects the impact of ARC symptoms on quality of life. Age-specific versions of the RQLQ were used. The RQLQ for patients aged 6–11 years consists of five dimensions: activity limitation, nasal symptoms, ocular symptoms, non-nasal/ocular problems, and other (20). The RQLQ for patients aged 12–17 years consists of six dimensions: activity limitation, nasal symptoms, ocular symptoms, non-nasal/ocular problems, practical problems, and emotional function (21). The RQLQ for patients aged ≥18 years consists of seven dimensions: activity limitation, nasal symptoms, ocular symptoms, non-nasal/ocular problems, sleep problems, practical problems, and emotional function (22). Each item was scored on a 7-point Likert scale (0 = no impairment, 6 = severe impairment). The overall RQLQ score was calculated as the sum of all item scores for each patient’s age-specific version (20–22). As the number of items differs across age groups, changes in RQLQ scores were analyzed only within groups. Additionally, to assess patient improvement over time, the change from baseline was calculated for each score at the 1, 2, and 3 years of SLIT time points.

### 2.4 Statistical analysis

Statistical analyses were performed using SPSS software (version 20.0; IBM Corp., Armonk, NY, USA), while graphs were generated using GraphPad Prism (version 8.0; GraphPad Software, La Jolla, CA, USA). Intergroup comparisons were conducted by the Mann–Whitney U test and intragroup comparisons of outcome measures across different time points (baseline, 1, 2, and 3 years) were performed using the Friedman test followed by adjusted post-hoc pairwise analyses using the Dunn-Bonferroni correction. A *p*-value < 0.05 was considered statistically significant.

## 3 Results

### 3.1 Patient characteristics

Of the 216 patients with ARC who agreed to SLIT, 66 patients did not complete the study (lost to follow-up: *n* = 35; poor adherence: *n* = 11; economic burden: *n* = 10; other reasons: *n* = 10). Consequently, complete data from the remaining 150 patients who completed the 3-year treatment were analyzed. There were 89 male (59.33%) and 61 female (40.67%) patients, with a mean age of 21.90 ± 14.33 years (range: 6–59 years). According to age-specific RQLQ requirements, patients were stratified into three groups: 6–11 years group (*n* = 40), 12–17 years group (*n* = 42), and ≥18 years group (*n* = 68). The baseline demographic and clinical characteristics of the study population are shown in Table 1.

**Table 1:** Patient characteristics.

Characteristics	SLIT group (*n* = 150)
Age	
Mean ± SD (year)	21.90 ± 14.33
Children (*n*, %)	60 (40.00%)
Adult (*n*, %)	90 (60.00%)
Gender	
Male (*n*, %)	89 (59.33%)
Female (*n*, %)	61 (40.67%)
Course (year)	
<3 (*n*, %)	75 (50.00%)
3–5 (*n*, %)	54 (36.00%)
6–10 (*n*, %)	8 (5.33%)
>10 (*n*, %)	13 (8.67%)
Baseline data scores (mean ± SD)	
TNSS	8.84 ± 1.35
TOSS	2.73 ± 0.99
TMS	2.00 ± 0.00
CSMRS	3.93 ± 0.35
Overall RQLQ	
6–11 years	56.50 ± 9.08
12–17 years	68.24 ± 10.80
≥18 years	77.21 ± 11.88

### 3.2 TNSS, TOSS, TMS, CSMRS, and VAS scores evaluation

**Figure 2:**
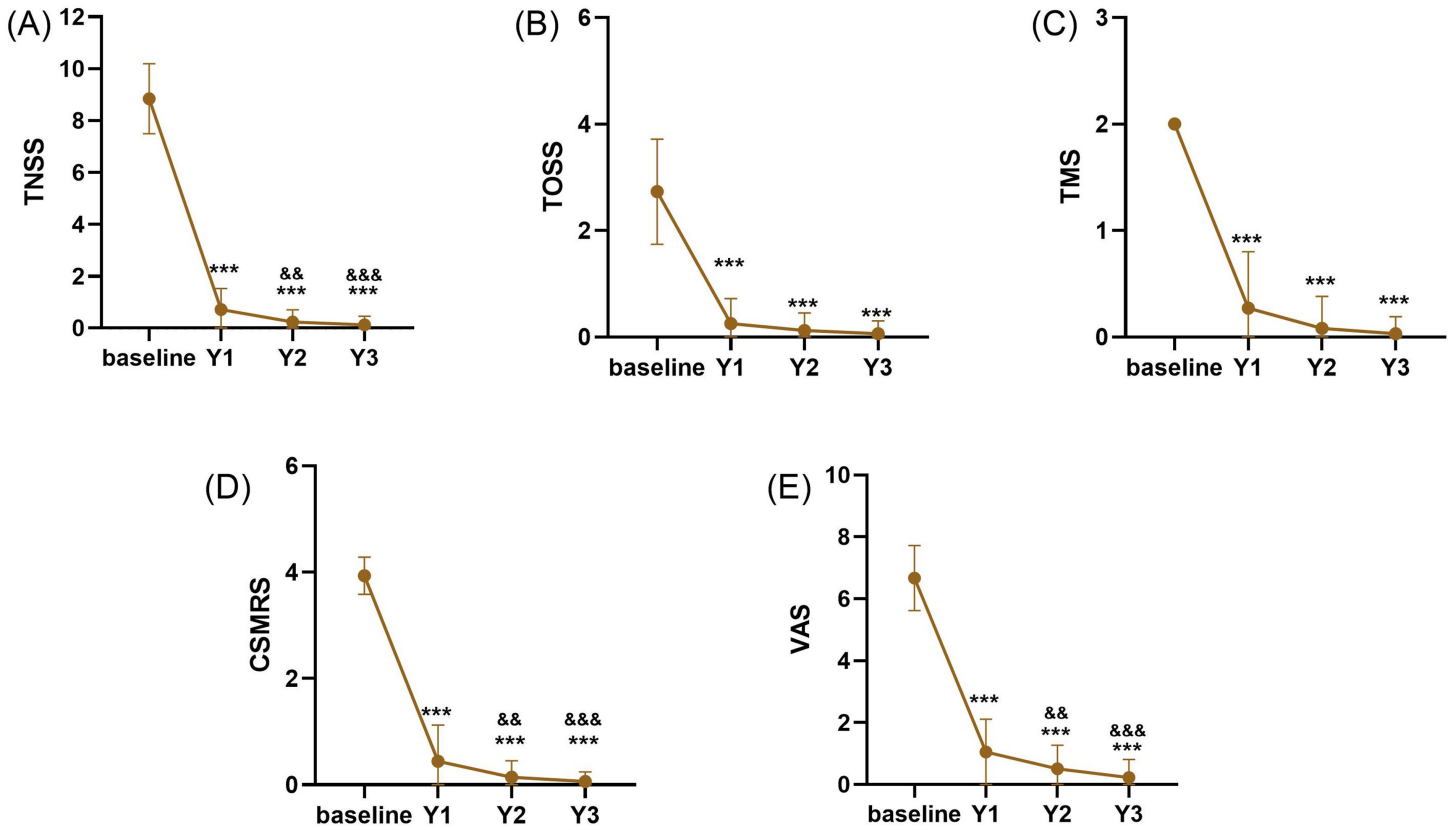
Clinical outcomes over 3 years of HDM-SLIT. **(A)** TNSS, **(B)** TOSS, **(C)** TMS, **(D)** CSMRS, and **(E)** VAS. ^*^indicates significant differences for all scores at 1, 2, and 3 years compared with baseline, ^***^p < 0.001. ^&^ indicate significant differences for the 2 and 3 years compared with the 1 year of HDM-SLIT, ^&&^*p* < 0.01; ^&&&^*p* < 0.001.

Compared with baseline, significant improvements in TNSS, TOSS, TMS, CSMRS, and VAS scores were observed after 1, 2, and 3 years of HDM-SLIT (all *p* < 0.001). Compared with the 1 year of HDM-SLIT, significant further improvements were found in TNSS, CSMRS, and VAS scores at both 2 (all *p* < 0.01) and 3 years (all *p* < 0.001) of HDM-SLIT treatment. Although no statistically significant differences were detected between the 2 and 3 years of HDM-SLIT for any of the clinical outcomes (all *p* > 0.05), all scores continued to show numerical improvements (TNSS: from 0.36 ± 0.66 to 0.19 ± 0.48; TOSS: from 0.24 ± 0.47 to 0.13 ± 0.33; TMS: from 0.08 ± 0.30 to 0.03 ± 0.16; CSMRS: from 0.14 ± 0.38 to 0.06 ± 0.22; VAS: from 0.51 ± 0.76 to 0.23 ± 0.58), suggesting a sustained trend toward further improvement (Figure 2).

### 3.3 RQLQ score evaluation

**Figure 3:**
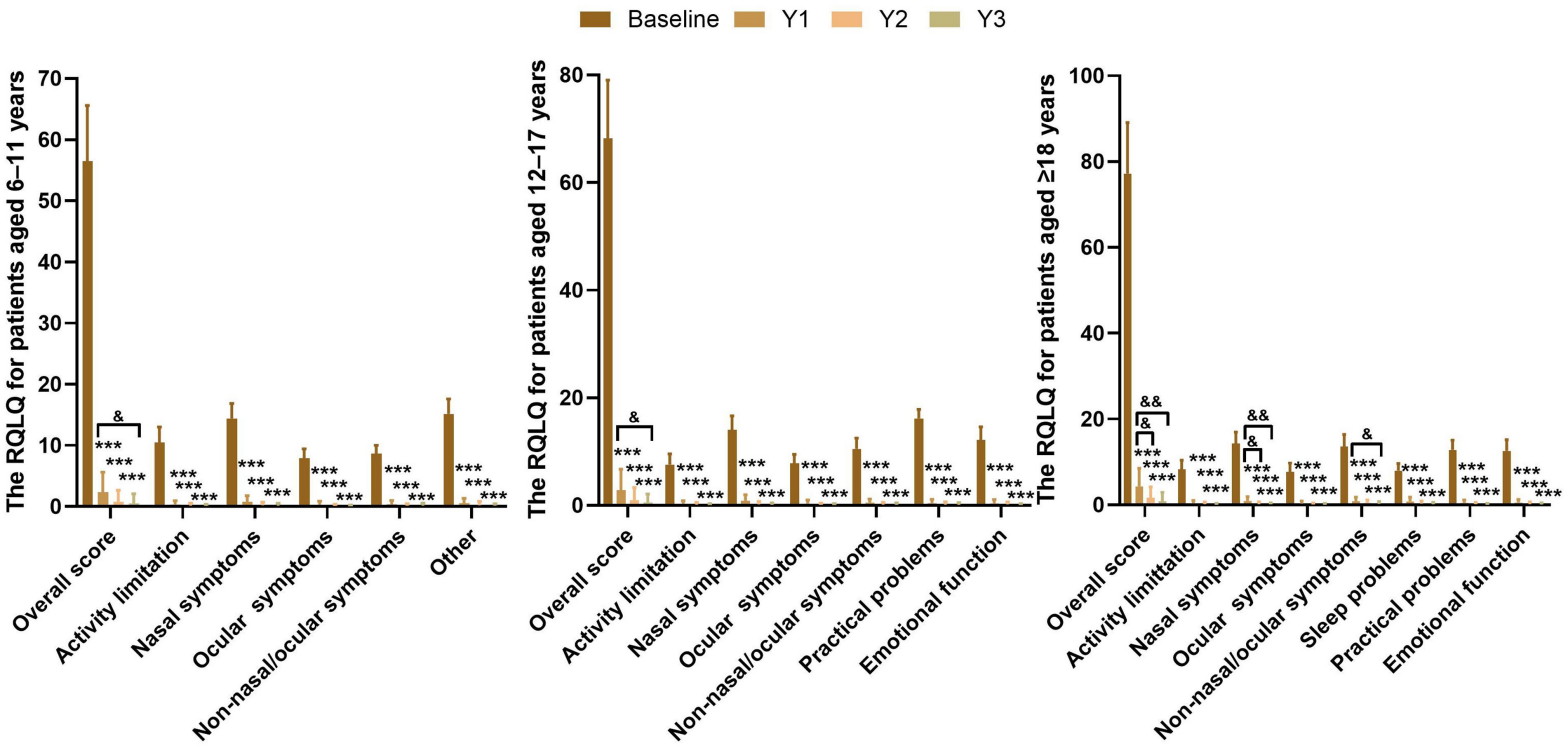
Changes in RQLQ scores and its domains over 3 years of HDM-SLIT. The RQLQ overall score and differences domains were assessed at baseline and after 1, 2, and 3 years of HDM-SLIT. ^*^ indicate significant differences at 1, 2, and 3 years of HDM-SLIT compared with baseline, ^***^p < 0.001. ^&^indicates significant differences at 2 and 3 years compared with the 1 year of HDM-SLIT, ^&^*p* < 0.05, ^&&^*p* < 0.01. Lower scores indicate better quality of life.

Compared with baseline, significant improvements in each RQLQ domain were observed after 1, 2, and 3 years of HDM-SLIT in all age groups (all *p* < 0.001). Compared with 1-year, significant improvements in overall score and nasal symptoms at 2 years of the ≥18 years group (all *p* < 0.05) and significant improvements in overall score at 3 years of all three age groups (6–11 years group: *p* < 0.05; 12–17 years group: *p* < 0.05; ≥18 years group: *p* < 0.01) were showed, as well as nasal symptoms (*p* < 0.01) and non-nasal/ocular problems (*p* < 0.05) at 3 years in the ≥18 years group (Figure 3).

### 3.4 Comparison of SLIT efficacy improvement

**Figure 4:**
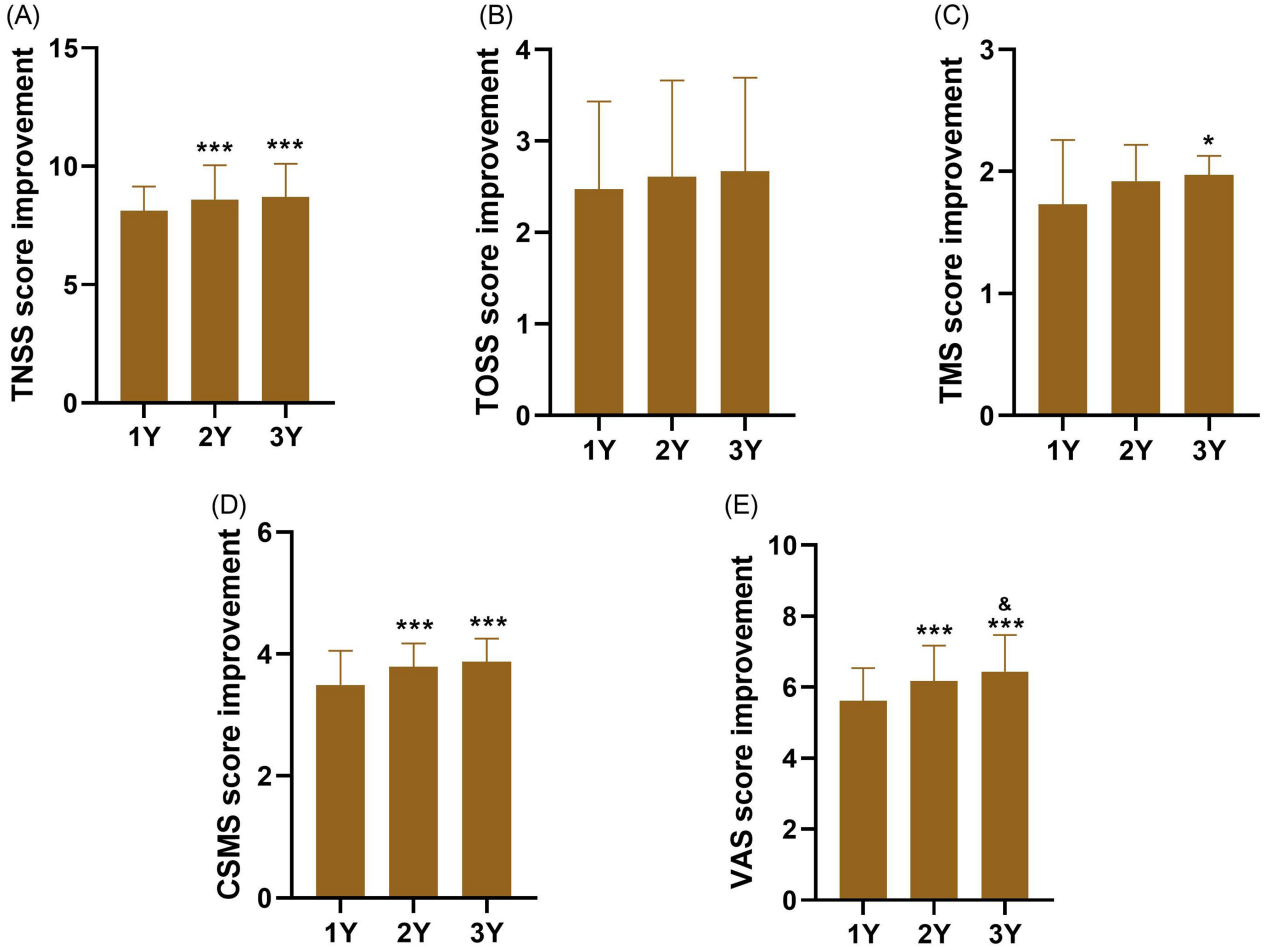
Clinical outcomes improvement at 1, 2, and 3 years of HDM-SLIT compared with baseline. **(A)** TNSS, **(B)** TOSS, **(C)** TMS, **(D)** CSMRS, and **(E)** VAS. ^*^indicate significant differences at 2 and 3 years compared with the 1 year of HDM-SLIT, ^*^p < 0.05, ^**^p < 0.01, and ^***^p < 0.001. ^&^indicate significant differences at 3 years compared with the 2 years of HDM-SLIT, ^&&^*p* < 0.01; ^&&&^*p* < 0.001.

We further evaluated the efficacy of HDM-SLIT by comparing the differences between baseline and 1-year, 2-year, and 3-year outcomes. Compared with the 1 year of SLIT, significant improvements in TNSS, CSMRS, and VAS scores were observed after 2 years of treatment (all *p* < 0.001). At 3 years, compared with the 1 year of SLIT, the TNSS (*p* < 0.001), TMS (*p* < 0.05), CSMRS (*p* < 0.001), and VAS scores (*p* < 0.001) all showed significant improvement. Furthermore, when comparing the 3 years with those at 2 years of SLIT, a significant further improvement was found in VAS scores (*p* < 0.05). Although no statistically significant differences were detected between the 2 and 3 years for TOSS score compared 1 year of SLIT (all *p* > 0.05), these scores continued to show numerical improvements (1 year: 2.49 ± 0.96; 2 years: 2.61 ± 1.05; 3 years: 2.67 ± 1.02), suggesting a sustained trend toward further ocular symptom relief (Figure 4).

### 3.5 Evaluation in different age group

**Figure 5:**
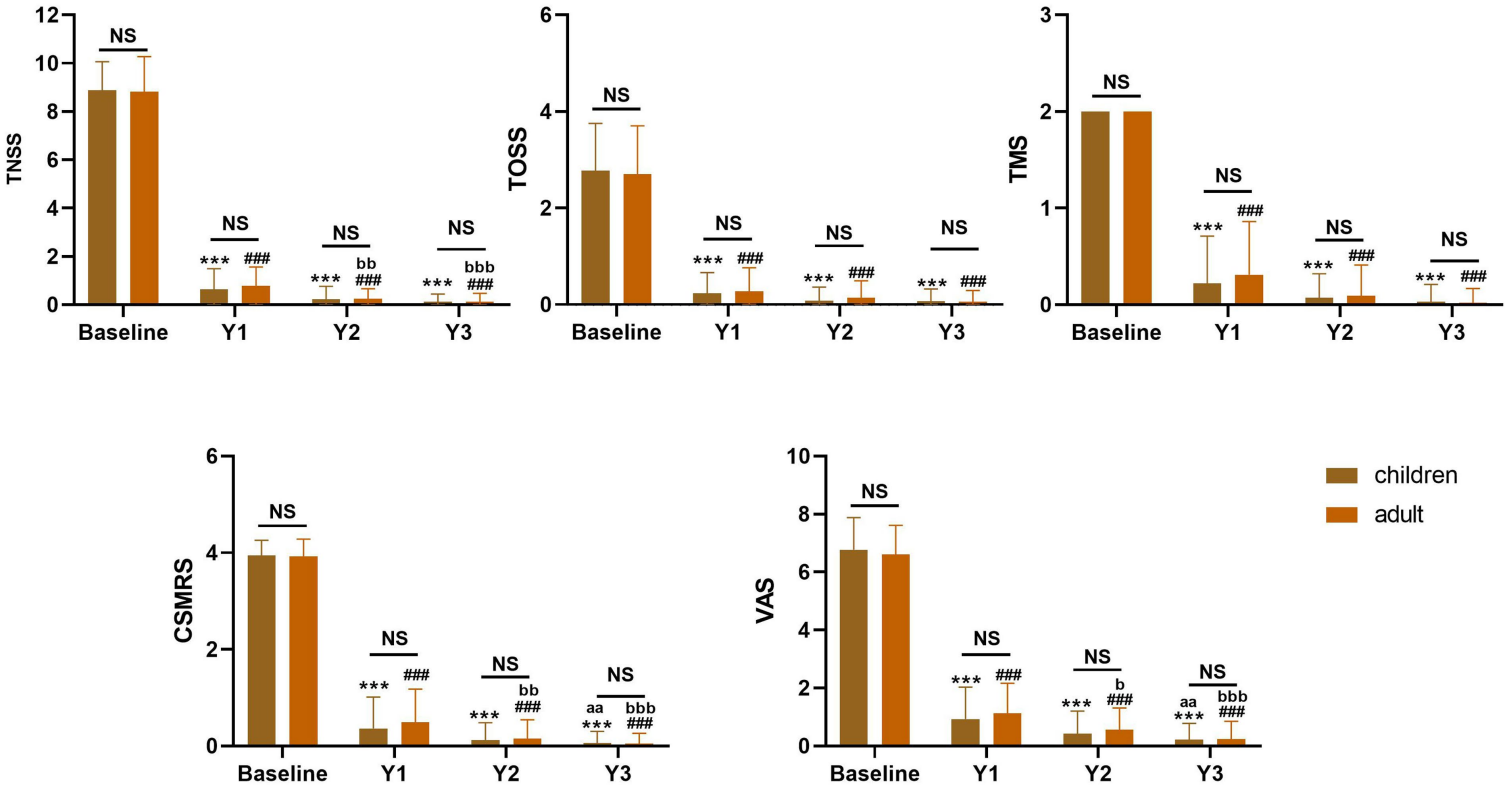
Clinical outcomes of **(A)** TNSS, **(B)** TOSS, **(C)** TMS, **(D)** CSMRS, and **(E)** VAS over 3 years of HDM-SLIT in the children and adult group. ^*^indicate significant differences for all scores at 1, 2, and 3 years compared with baseline in the children group, ^***^ *p* < 0.001. ^#^indicate significant differences for all scores at 1, 2, and 3 years compared with baseline in the adult years group, ^###^ *p* < 0.001. ^a^indicate significant differences for the 2 and 3 years compared with the 1 year of HDM-SLIT in the children group, ^aa^*p* < 0.01; ^&&&^*p* < 0.001. ^b^indicate significant differences for the 2 and 3 years compared with the 1 year of HDM-SLIT in the adult group, ^b^*p* < 0.05; ^bb^*p* < 0.01, ^bbb^*p* < 0.001. NS, no significant difference.

In addition, we conducted further subgroup comparisons between the children group (n = 60) and adult group (n = 90). In this study, no significant differences were observed between the two groups in any baseline clinical outcomes (TNSS, TOSS, TMS, CSMRS, and VAS; all *p* > 0.05). Furthermore, there were no significant differences between the two groups in any clinical outcomes at 1, 2, and 3 years of HDM-SLIT. In the children group, compared with the 1 year of HDM-SLIT, there were significant difference in the CSMRS and VAS scores at 3 years (all *p* < 0.01). In the adult group, compared with the 1 year of HDM-SLIT, the TNSS (*p* < 0.01), CSMRS (*p* < 0.01), and VAS (*p* < 0.05) scores showed significant differences at 2 years of HDM-SLIT, and these improvements were maintained at 3 years of HDM-SLIT (all *p* < 0.01, Figure 5).

### 3.6 Safety

During the period of the whole study, 8% (12/150) of patients reported AEs with 11 in the up-dosing phase and 1 in the maintenance phase. The primary AEs were itchy sense in the mouth or tongue, as well as some aggravating allergic symptoms. These AEs could be relieved by themselves or through the use of symptomatic medication. Additionally, no severe AEs were reported during the entire study.

## 4 Discussion

Allergic diseases, particularly AR with or without AC, represent a significant global health burden, affecting millions of individuals across different age groups and causing substantial impairment in quality of life and productivity (14, 23). Among the various treatment modalities, SLIT has emerged as a safe and effective option and is widely used in clinical practice. In China, HDM-SLIT has been applied for 20 years, with numerous clinical trials demonstrating its efficacy and safety in both children and adults with AR, with or without AC (6, 13). Multiple studies have shown that SLIT treatment can significantly improve the symptom of patients, reduce the use of medications, and lower the VAS score (15–17, 24). Our study also observed that after 3 years of HDM-SLIT, the TNSS, TMS, CSMRS, and VAS scores of the patients all showed significant improvement, and no severe AEs were reported throughout the study. These findings provide new evidence supporting the efficacy and safety of SLIT.

However, despite the frequent coexistence of ocular symptoms in patients with AR, the specific efficacy of SLIT on ocular manifestations has not been adequately characterized (12, 13), as most studies have either prioritized nasal outcomes or employed composite rhinoconjunctivitis endpoints. A systematic review indicated that SLIT can significantly reduce total ocular symptom scores and individual scores for itching, redness, and tearing (12). A prospective study has shown that SLIT treatment not only improves the TNSS and TMS scores of patients, but also lower their TOSS scores (25). Our three-year follow-up study not only corroborates previous findings on SLIT efficacy (12, 13, 25) but also provides novel insights by demonstrating the sustained and progressive nature of ocular symptom improvement—an aspect rarely addressed in prior research. The TOSS score was significantly improved at all time points compared to baseline. Moreover, the magnitude of improvement increased further during the later stages of treatment. This suggests that the benefits of HDM-SLIT on ocular symptoms are not only immediate but also accumulate over time, providing evidence supporting the clinical application of SLIT for allergic conjunctivitis. This long-term and progressive improvement pattern is a key highlight of our study, setting it apart from previous research and offering new perspectives on the management of ocular symptoms in AR patients. Accumulating evidence indicates that SLIT induces systemic immunomodulation, including the generation of allergen-specific IgG4 blocking antibodies, which competitively inhibit IgE binding to mast cells and basophils, thereby reducing the release of histamine and other inflammatory mediators responsible for ocular itching and redness (27, 28). Additionally, SLIT promotes the expansion of regulatory T cells (Tregs), which suppress Th2-driven allergic responses through the secretion of anti-inflammatory cytokines such as interleukin-10 and transforming growth factor-β (27, 28). These immunological changes accrue over time, leading to progressive clinical improvement. Therefore, the shared Th2 inflammatory pathway, naso-ocular reflex, and desensitization mechanism may explain why SLIT alleviates both nasal and ocular symptoms (1, 4).

Health-related quality of life is increasingly recognized as a crucial outcome measure in allergic diseases, as it provides a comprehensive assessment of the impact of the disease on patients’ daily functioning, emotional well-being, and overall health status (26, 27). This study employed age-specific RQLQ and, for the first time, performed a stratified analysis of treatment outcomes across three age groups (6–11 years, 12–17 years, and ≥18 years groups), within a single study. The results showed significant and sustained improvements across all RQLQ domains in all age groups. This finding has important clinical implications: First, it confirms that HDM-SLIT provides clinically meaningful quality-of-life benefits across different age groups. Second, our study observed that the most pronounced improvement in quality of life occurred within the first year of treatment and was maintained over the subsequent two years, with some parameters (e.g., overall score) showing further optimization. Moreover, in the adult group, the overall score, nasal symptoms, and non-nasal/ocular symptom scores showed significant improvement. We consider that this might be related to the larger number of participants in the adult group. The studies conducted by Huang et al. (24), Schuster et al. (28), and Blaiss et al. (29) all demonstrated that SLIT can improve the quality of life of patients, which is highly consistent with our findings.

An important observation from our long-term follow-up is the progressive nature of clinical improvement with continued SLIT treatment, which aligns with accumulating evidence on the relationship between treatment duration and therapeutic outcomes. Several studies have demonstrated that extended SLIT courses confer greater clinical benefits. Lin et al. (15) found that patients completing 3 years of SLIT achieved the highest proportion of medication withdrawal compared with those receiving shorter durations, suggesting that 3-year SLIT is more efficacious than 1- or 2-year courses. Similarly, Yang et al. (17) reported that patients receiving 3–4 years of SLIT showed significant improvements compared with those receiving only 2 years of treatment, with the benefits sustained even one year after treatment discontinuation. Notably, their study observed that 3- and 4-year courses yielded comparable outcomes, indicating that 3 years may represent an optimal treatment duration for achieving maximal clinical benefit. Our findings extend these observations by demonstrating that the therapeutic effects of HDM-SLIT continue to accumulate beyond the first year of treatment. Compared with the 1-year results, we observed numerical improvements continuing across all outcomes between 2 and 3 years. The long-term efficacy of SLIT has been further corroborated by Chen et al. (16), who demonstrated that 3-year SLIT combined with pharmacotherapy remained effective for at least 3 consecutive years after treatment cessation in children with AR, with no significant differences between scores at 3-year and 6-year follow-ups. Collectively, these findings support current recommendations for a minimum of three years of SLIT to achieve optimal and sustained disease-modifying effects (13).

Furthermore, subgroup analysis according to age revealed no significant differences in clinical outcomes between the two age groups after SLIT treatment. However, the children group exhibited slightly better efficacy compared with the adult group, although the smaller sample size in the younger group may have limited the ability to detect smaller differences. This finding is consistent with a recent study by Huang et al. (24), who reported that house dust mite sublingual immunotherapy seems more effective in children than in adolescents and adults with allergic rhinitis. We consider that this difference may be attributed to age-related changes in immune system plasticity. The immune system exhibits greater plasticity in childhood, with a more pronounced capacity for regulatory T-cell induction and immune deviation following allergen immunotherapy (27, 28). The EAACI guidelines also emphasize that early initiation of AIT in children may induce more sustained immune tolerance due to this heightened plasticity (8). Chen et al. (16) demonstrated that 3-year SLIT in children provided sustained benefits for at least 3 years after treatment cessation, further supporting the long-term value of early intervention. Collectively, these findings underscore the importance of early intervention in modifying the natural course of allergic diseases (8, 15, 17).

However, the current study has several limitations. First, the single-center, open-label design without a placebo control may introduce potential bias and limit the generalizability of our findings, although the sustained and progressive improvements over 3 years argue against a mere placebo effect. Second, we did not assess immunological parameters, which could provide mechanistic insights into the clinical responses observed. In the future, we will consider further exploring the related mechanisms in studies with placebo controls.

## 5 Conclusion

This 3-year retrospective study demonstrates that HDM-SLIT yields significant, sustained, and progressive improvements in both clinical outcomes and quality of life in patients with ARC. Throughout the 3-year treatment period, no serious systemic adverse events were reported. By isolating ocular symptoms from composite rhinoconjunctivitis endpoints, we provide supportive evidence that HDM-SLIT significantly alleviates ocular manifestations, with benefits accumulating over time and persisting throughout the 3-year treatment period. Furthermore, age-stratified analysis using disease-specific RQLQ confirms that these therapeutic benefits extend across patients of all ages. These findings support HDM-SLIT as an effective and safe therapy for ARC and underscore the importance of dedicated ocular symptom assessment in both clinical practice and future research.

## Data Availability

The raw data supporting the conclusions of this article will be made available by the authors, without undue reservation.
